# Finding Key Nodes in Complex Networks Through Quantum Deep Reinforcement Learning

**DOI:** 10.3390/e27040382

**Published:** 2025-04-03

**Authors:** Juechan Xiong, Xiao-Long Ren, Linyuan Lü

**Affiliations:** 1 Institute of Fundamental and Frontier Sciences, University of Electronic Science and Technology of China, Chengdu 611731, China; juechanxiong@std.uestc.edu.cn; 2 Yangtze Delta Region Institute (Huzhou), University of Electronic Science and Technology of China, Huzhou 313001, China; 3 School of Cyber Science and Technology, University of Science and Technology of China, Hefei 230026, China

**Keywords:** vital node identification, quantum algorithm, reinforcement learning, complex networks

## Abstract

Identifying key nodes in networks is a fundamental problem in network science. This study proposes a quantum deep reinforcement learning (QDRL) framework that integrates reinforcement learning with a variational quantum graph neural network, effectively identifying distributed influential nodes while preserving the network’s fundamental topological properties. By leveraging principles of quantum computing, our method is designed to reduce model parameters and computational complexity compared to traditional neural networks. Trained on small networks, it demonstrated strong generalization across diverse scenarios. We compared the proposed algorithm with some classical node ranking and network dismantling algorithms on various synthetical and empirical networks. The results suggest that the proposed algorithm outperforms existing baseline methods. Moreover, in synthetic networks based on Erdős–Rényi and Watts–Strogatz models, QDRL demonstrated its capability to alleviate the issue of localization in network information propagation and node influence ranking. Our research provides insights into addressing fundamental problems in complex networks using quantum machine learning, demonstrating the potential of quantum approaches for network analysis tasks.

## 1. Introduction

A large number of complex systems in nature can be simplified and described by various networks [1], such as social relationship networks [2], scientific collaboration networks [3], the World Wide Web (WWW) [4], citation networks [5], food chain networks [6], and protein–protein interaction networks [7]. Under the name of network science, unraveling complexity with networks has became a vibrant research field for the past decades. Research on the structure and function of these networks has revealed universal characteristics across different systems, such as the small-world phenomenon, the power-law distribution of node degrees, and the community structures within networks. An important research focus in network science was ranking nodes according to their influence, which has had numerous practical applications [8,9,10]. Important nodes, often referred to as critical nodes, are those that significantly influence the structure and functionality of networks [11,12,13,14]. Although the number of these critical nodes is typically small, their impact can rapidly propagate through the network, causing cascading disruptions that affect a large portion of the system [15,16]. This phenomenon underscores the necessity of accurately ranking node significance and identifying critical nodes to enhance our understanding of network robustness and to inform strategies for maintaining system integrity.

The complexity of graph structures arises from the non-Euclidean nature of graph-structured data [17]. A potential solution for handling complex patterns lies in embedding techniques, which learn graph representations in low-dimensional Euclidean spaces [18,19,20]. Graph embedding techniques embed high-dimensional and sparse network representations into low-dimensional dense vector spaces while preserving the original network’s topological information. Once low-dimensional representations are learned, many graph-related tasks, such as node classification and link prediction, can be performed effectively [20]. Despite the successes of existing embedding methods, many earlier approaches were constrained by shallow learning mechanisms [20,21], limiting their ability to capture more intricate patterns inherent in graphs. While a diverse range of deep learning methods, such as graph transformers, has been developed, these approaches often face significant computational overheads and rely on domain-specific assumptions, which may limit their generalizability and scalability in diverse graph-based applications [22].

On the other hand, identifying an optimal series of critical nodes in general graphs to optimize nontrivial and hereditary connectivity measures is often an NP-hard problem [23,24,25]. Deep learning has demonstrated its efficacy in numerous applications. Inspired by recent advances in deep learning techniques for solving combinatorial optimization problems [26,27,28], this study integrated deep learning with complex network analysis to address the critical node identification problem. However, as deep learning models became increasingly complex, the number of parameters required to represent these models grew significantly. This dramatic expansion in parameter space poses substantial challenges in terms of computational cost and model generalization—issues that are conceptually related to the difficulties encountered in high-dimensional spaces.

In summary, reinforcement learning (RL) offers a robust framework for sequential decision making under uncertainty, and its deep variants have proven effective in approximating complex value functions and policies [29,30]. In our work, we leverage RL to iteratively optimize node ranking based on cumulative rewards derived from network dismantling tasks. While classical RL methods have shown success across various domains, their capacity to capture the intricate, nonlinear interdependencies inherent in complex networks can be limited.

Quantum deep reinforcement learning (QDRL) extends this framework by incorporating quantum computing principles, such as superposition and entanglement, to potentially process high-dimensional state spaces more efficiently. Recent surveys and studies in quantum reinforcement learning [31,32] indicate that QDRL may offer a novel computational advantage, particularly in environments with complex dynamics. Furthermore, advances in offline RL [33,34] underscore the importance of developing robust learning algorithms under practical constraints.

Our proposed method is presented as a proof-of-concept that demonstrates the feasibility of employing quantum algorithms for the node ranking problem. By situating our approach within the context of the existing literature, we highlight both its theoretical foundation and its potential for future scalability. As quantum hardware continues to advance, we anticipate that the scalability and efficiency of QDRL will further improve, potentially offering advantages over classical methods in the analysis of large-scale complex networks.

The remainder of this paper is organized as follows. Section 2 provides an overview of the background knowledge, including Q-learning-based reinforcement learning methods, the fundamentals of quantum computing, and the components of variational quantum circuits. In Section 3, we present the algorithm design for identifying critical nodes in networks using quantum reinforcement learning. Section 4 details the experiments conducted on both real-world and synthetic networks, along with an analysis of the effectiveness and advantages of the proposed method. Finally, Section 5 concludes the paper and discusses potential future directions.

## 2. Quantum Deep Reinforcement Learning

This section introduces the fundamental techniques employed in the proposed method, including the Double Deep Q-Network, the basic concepts of quantum computing, and the principles and components of variational quantum circuits.

### 2.1. Double Deep Q-Network

Reinforcement learning addresses the problem of how an agent can maximize its cumulative reward within a complex and uncertain environment. During the training process, the agent interacts with the environment by observing a state $s_{t}\in S$ and then selecting an action $a_{t}\in A$ according to a policy π:S→A (i.e., $a_{t}=\pi \left(s_{t}\right)$). The environment then transitions to a new state according to $s_{t+1}=f\left(s_{t},a_{t}\right),$ and returns a reward $r_{t}=r\left(f(s_{t},a_{t})\right),$ where r:S→R is a transition of the state. The discount factor γ∈(0,1] is a hyperparameter that determines the present value of future rewards.

In Q-learning, the agent learns a Q-value, which evaluates the expected cumulative reward starting from a given state–action pair (s,a), according to the following policy π as (1) until the end of the episode. This $Q^{\pi }\left(s,a\right)$ value is updated iteratively to optimize the agent’s decision-making process [35,36].

(1) $$Q^{\pi }\left(s,a\right)=E_{\pi }\left[\sum_{k=0}^{\infty }\gamma^{k}\;r\left(s_{t+k},\pi \left(s_{t+k}\right)\right)\;|\;s_{t}=s,\;a_{t}=a\right].$$

Here, the expectation $E_{\pi }$ is taken over all possible future state trajectories and any stochasticity in the environment and/or policy. Note that $Q^{\pi }\left(s,a\right)$ depends on the initial action *a* because different actions lead to different subsequent state trajectories and reward sequences. The optimal Q-function is defined as $Q^{*}\left(s,a\right)=\max_{\pi }Q^{\pi }\left(s,a\right)$ and by selecting the action with the highest Q-value at each step. Thus, the objective of Q-learning is to accurately estimate $Q^{*}\left(s,a\right)$. The objective of Q-learning is to estimate the optimal Q-function [29]. To ensure sufficient exploration of the environment by the agent, a commonly used approach during training is the ϵ-greedy strategy. This strategy involves selecting actions randomly with a probability of ϵ while choosing the action with the highest Q-value with a probability of 1−ϵ. It is important to note that the Q-value reflects the cumulative reward of not only the immediate action but also of all subsequent actions determined by π [30,36]. The agent updates the Q-function through interactions with the environment, following the equation below: [37]:

(2) $$Q\left(s_{t},a_{t}\right)\leftarrow Q\left(s_{t},a_{t}\right)+\alpha \left[r_{t}+\gamma \max_{a\in A}Q\left(s_{t+1},a\right)-Q\left(s_{t},a_{t}\right)\right],$$

where α is the learning rate, $r_{t}$ is the reward at time step *t*, and γ represents the discount factor, reflecting the significance of future rewards. This update is applied iteratively as the agent interacts with the environment, and under standard conditions, it converges to the optimal Q-function $Q^{*}\left(s,a\right)$ [37,38].

This paper proposes a quantum circuit design approach based on the Double Deep Q-Network (DDQN) and experience replay techniques to enhance training stability [30]. Integrating DDQN’s improved action selection mechanism and experience replay’s efficient memory utilization provides a more robust training process for quantum circuits.

### 2.2. Quantum Computing

Quantum computing [39] is a novel computational paradigm that leverages fundamental principles of quantum mechanics, such as superposition, interference, and entanglement, to process quantum information units. The basic unit of quantum information is the qubit, which, unlike a classical bit, can exist in a superposition of 0 and 1. Using Dirac notation, any quantum state can be expressed as

(3) $$|\psi \rangle =\alpha |0\rangle +\beta |1\rangle ,\;\mathrm{with}\;\alpha ,\beta \in {C,|\alpha |}^{2}+{\left|\beta \right|}^{2}=1,$$

where |0〉 and |1〉 denote the computational basis states in a two-dimensional Hilbert space. When a measurement is performed on |ψ〉, it collapses to either |0〉 or |1〉, with probabilities ${\left|\alpha \right|}^{2}$ and ${\left|\beta \right|}^{2}$, respectively. This property of superposition underlies the potential computational advantages of quantum computers relative to classical ones. Moreover, quantum gates *U* act on qubits through unitary transformations, which are analogous to the logic gates used in classical computing.

(4) $$|\psi^{\prime }\rangle =U|\psi \rangle .$$

A quantum system’s state can be transformed through sequential applications of unitary operators *U* before measurement. These operators act as linear transformations in complex Hilbert space and are characterized by the properties $U^{†}U=UU^{†}=I$, ensuring both reversibility and norm preservation of the quantum state vector. Each unitary operation represents a coherent manipulation of the system’s quantum state while maintaining quantum superposition.

Classical computers are represented by circuits consisting of wires and logic gates. Analogously, quantum computers can be represented using quantum circuits comprising wires and quantum gates. In a quantum circuit, each wire corresponds to a qubit that carries quantum information, while quantum gates transform quantum states.

### 2.3. Variational Quantum Circuits

**Figure 1:**
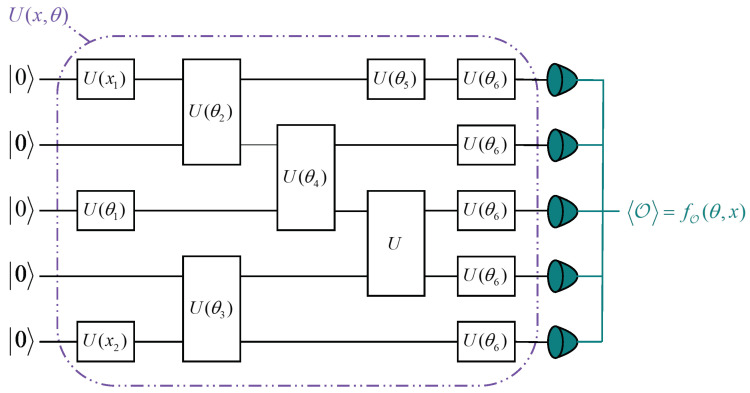
Schematic diagram of a VQC. Each wire corresponds to one qubit, and each box on a wire represents a single-qubit gate. Boxes spanning multiple wires represent multi-qubit gates. The number of wires in the circuit corresponds to the number of qubits in the system. The input data are represented by *x*, the adjustable parameters by θ, and the quantum gates by *U*. All elements are initialized to $|0\rangle$, with the final layer performing measurement operations.

Variational quantum algorithms (VQAs) are an effective approach to implementing algorithms on Noisy Intermediate-Scale Quantum (NISQ) computers [40], as they are particularly well suited for systems with a limited number of qubits, the presence of noise, and constrained coherence times [41]. Variational quantum circuits (VQCs) are a set of quantum gates operating on multi-qubit quantum systems [41,42]. Their fundamental operating principle lie in the combination of parameterized quantum circuits, with parameters adjusted by classical optimizers to achieve the desired results, while being evaluated in each optimization step [43]. VQCs were first introduced in the context of the Variational Quantum Eigensolver (VQE) [44] and have since become a major research focus in quantum machine learning [45,46,47]. An example of a VQC with five qubits is shown in Figure 1. To provide a more detailed description of the entire VQC, suppose we have some objective function f(θ,x) of a quantum circuit,

(5) $$f\left(\theta ,x\right)=\langle 0|U^{†}\left(\theta ,x\right)\hat{M}U\left(\theta ,x\right)|0\rangle ,$$

where |0〉 is the initial state, and $\hat{M}$ denotes the observable, and the parameterized gate U(θ,x) is

(6) $$U\left(\theta ,x\right)=e^{-i\theta Gx}.$$

Here, *G* is the Hermitian generator of the gate [48].

Thus, a VQC typically consists of three main components:

(1) Initialization of Quantum States: The initial quantum state is prepared by setting all qubits to $|0\rangle$.
(2) Parameterized Quantum Circuit: The parameterized quantum circuit (PQC) consists of input parameters *x* and variational parameters θ, as illustrated in Figure 1. PQCs are trained by querying quantum devices through classical optimization algorithms. The input data *x* are used for information embedding, mapping classical data *x* and θ to quantum states $U\left(x,\theta \right)|0\rangle$ in the Hilbert space through parameterized quantum gates [49,50]. Similar to the weights in neural networks, variational parameters are randomly initialized before training. During the iterative process, the variational parameters θ are adjusted using appropriate methods to optimize the loss function. For instance, in the context of supervised machine learning, the loss function L can be minimized by performing gradient descent over $\nabla_{\theta }L$. Several analytical and numerical approaches have been developed to compute the gradients of quantum circuits with respect to their parameters [51,52,53]. In this study, we employed the parameter-shift rule for gradient computation. A parameterized quantum circuit can be regarded as a function operating on *N* qubits over *L* layers. For a given layer *l*, it can be represented as a set of parallel single-qubit rotation gates:
  (7) $$U^{l}\left(\theta^{l},x^{l}\right)=\underset{j=1}{\overset{N}{⊗}}\;U_{j}^{l}\left(\theta_{j}^{l},x_{j}^{l}\right).$$
  These single-qubit rotation gates can be expressed as $U_{j}^{l}\left(\theta_{j}^{l},x_{j}^{l}\right)=e^{-iaG_{j}^{l}\theta_{j}^{l}x_{j}^{l}}$, where $G_{j}^{l}$ is a linear combination of Pauli operators, and *a* is a real constant. $G_{j}^{l}$ can be represented as a Hermitian matrix with two eigenvalues, $e_{0}$ and $e_{1}$ [48]. Owing to the properties of the exponential function, the derivative of $U_{j}^{l}\left(\theta_{j}^{l},x_{j}^{l}\right)$ can be written as
  (8) $$\frac{\partial U_{j}^{l}\left(\theta_{j}^{l},x_{j}^{l}\right)}{\partial \theta_{j}^{l}}=-iaG_{j}^{l}x_{j}^{l}e^{-iaG_{j}^{l}\theta_{j}^{l}x_{j}^{l}}=-iaG_{j}^{l}x_{j}^{l}U_{j}^{l}\left(\theta_{j}^{l},x_{j}^{l}\right).$$
  Therefore, after the measurement operation, the derivative of the entire circuit can be expressed as
  (9) $$\frac{\partial f(\theta ,x)}{\partial \theta }=\langle 0|\left(\frac{\partial U^{†}\left(\theta ,x\right)}{\partial \theta }\right)\hat{M}\;U\left(\theta ,x\right)|0\rangle +\langle 0|U^{†}\left(\theta ,x\right)\hat{M}\left(\frac{\partial U(\theta ,x)}{\partial \theta }\right)|0\rangle .$$
  where $\hat{M}$ denotes the observable, and $U\left(\theta ,x\right)=\prod_{l=1}^{L}U^{l}\left(\theta^{l},x^{l}\right)=\prod_{l=1}^{L}{⊗}_{j=1}^{N}U_{j}^{l}\left(\theta_{j}^{l},x_{j}^{l}\right)$.
(3) Measurement Operations: The measurement operation involves measuring the expectation value of the observable $\hat{M}$, which is composed of one or more qubits. Typically, the loss function for a given task is defined by the expectation values $f_{\hat{M}}\left(\theta ,x\right)=\langle \hat{M}\rangle =\langle 0|U^{†}\left(\theta ,x\right)\hat{M}U\left(\theta ,x\right)|0\rangle$ of one or more VQCs. These expectation values can then serve as inputs for classical post-processing.

## 3. Methodology

We employed VQAs in reinforcement learning to identify key players in networks. Considering the complexity of mapping graph structures to quantum states, we combined message-passing-based graph neural network algorithms [54,55] to encode graphs into quantum states in Hilbert Space. These quantum graph states were then used as inputs for quantum reinforcement learning algorithms. By adjusting the parameters of the quantum gates in the VQAs based on the measurement results at the output, we trained the model on synthetic networks.

In our designed quantum reinforcement learning framework, the architecture primarily comprised encoder and decoder components. The encoder component mapped the network structure onto quantum circuits using a quantum graph convolutional network. This part aggregated neighborhood information on the quantum circuits, encoding the graph into quantum states while preserving the original graph structure as much as possible to facilitate subsequent processing using quantum computational methods.

The output of the encoder component served as the input to the decoder component. The decoder component used VQCs as function approximators for the Q-function in reinforcement learning. Apart from the approximator structure, other mechanisms were similar to those in DDQN: employing a target Q-network for delayed updates, using a greedy strategy to determine the agent’s next action, and performing experience replay to sample and train the Q-network based on VQCs.

**Figure 2:**
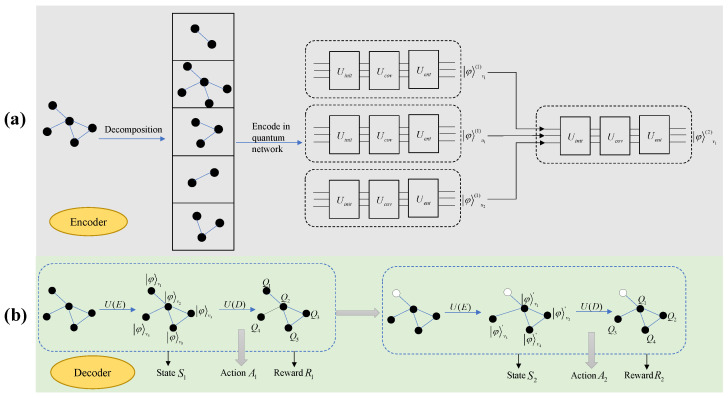
Model framework diagram. (**a**) illustrates the processing workflow of the encoder, which primarily encodes network data into quantum states. (**b**) depicts the decoder in a two-step Markov process.

The overall model framework is illustrated in Figure 2.

### 3.1. Encoder

Given the limited number of available qubits in current quantum systems, we implemented a graph partitioning strategy prior to training. For a graph *G* with *n* nodes, we decomposed it into *n* subgraphs, where each subgraph comprises node *i* and its first-order neighbors. During training on synthetic graphs, we utilized node *i*’s degree centrality, betweenness centrality, and other topological metrics as initial node features. For evaluation on real-world networks, we employed the intrinsic node features instead.

**Figure 3:**
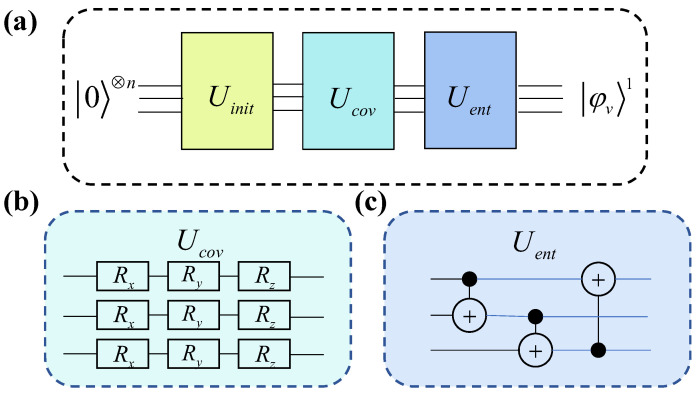
Quantum circuit diagram for the encoder aggregating first-order neighbor information. (**a**) illustrates the overall process of encoding first-order neighbors, which is divided into three components: $U_{\mathrm{init}}$, $U_{\mathrm{cov}}$, and $U_{\mathrm{ent}}$. ${|\varphi_{v}\rangle }^{1}$ in (**a**) denotes the quantum state representation of node *v* after aggregating information from its first-order neighbors. $U_{\mathrm{init}}$ encodes the feature vector of a node into the rotation gates of the quantum circuit. $U_{\mathrm{cov}}$, as shown in (**b**), encodes the parameters $U_{1}$ and $U_{2}$ into the node *v* and its neighboring node *u*, respectively. $U_{\mathrm{ent}}$, in (**c**), generates entanglement among all nodes.

The encoder mapped graph data into a Hilbert space amenable to quantum computation by encoding network nodes into quantum states while preserving the original graph’s neighborhood information. This was implemented through a multi-layer message-passing neural network constructed on quantum circuits to aggregate neighboring information. The mathematical formulation is as follows:

(10) $${|\varphi_{v}\rangle }^{t}=U_{1}{|\varphi_{v}\rangle }^{t-1}⊗\left(\underset{u\in N(v)}{⊗}\;U_{2}{|\varphi_{u}\rangle }^{t-1}\right),$$

where ${|\varphi_{v}\rangle }^{t}$ denotes the quantum state representation of node *v* at layer *t*, while $U_{1}$ represents the quantum gate parameters for node *v*. Similarly, ${|\varphi_{u}\rangle }^{t-1}$ refers to the quantum state features of *v*’s neighboring nodes *u* at layer t−1, and $U_{2}$ represents the quantum gate parameters employed by node *u*. The operator ⊗ signifies the tensor product operation. Parameters $U_{1}$ and $U_{2}$ jointly constitute the trainable parameters of the encoder component. Figure 3 illustrates the quantum circuit for aggregating first-order neighbor information in the encoder.

In Figure 3, each line represents a node. The circuit in the $U_{\mathrm{init}}$ component encodes node features into the rotation parameters of quantum gates, corresponding to the initial state of the nodes. $R_{X}$, $R_{Y}$, and $R_{Z}$ denote quantum gates that perform rotations on the X, Y, and Z axes, respectively. The rotation parameter corresponding to node *v* is $U_{1}$, and that of its neighboring node *u* is $U_{2}$. The quantum gates used in $U_{\mathrm{ent}}$ are CNOT gates, which entangle the nodes within the system. Figure 3 represents a quantum system corresponding to a subgraph composed of node *v* and its first-order neighbors. The output of this system is the quantum state representation of node *v* after aggregating the information from its first-order neighbors. The quantum state obtained after aggregating the first-layer information serves as the input for constructing the second-layer aggregation circuit. By iterating this process, the quantum state representation of a node embedding that aggregates information up to the *k*-hop neighborhood can be obtained.

To capture as much global information from the graph as possible, a global node was introduced to obtain the quantum state representation of the entire graph. A new global node was created that connects to all nodes in the graph while ensuring that the global node was not included in the neighbor sets of other nodes. The global node aggregated its neighbors following the process outlined in Figure 3. The output after aggregating multi-layer neighbors for the global node was used as the quantum state representation of the entire graph, corresponding to the state *S* in the decoder design.

**Figure 4:**
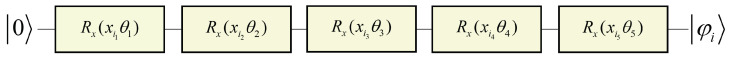
Quantum circuit diagram for the mapping component.

The $U_{\mathrm{init}}$ in Figure 3 is responsible for calculating the parameters of quantum rotation gates that map nodes onto the quantum circuit, representing the initialization of nodes. The steps are as follows:

(1) Randomly initialize the rotation parameter vector $\vec{\theta }$, with the same dimension as the initial features of the nodes. Let the initial feature of node *v* be $\vec{x_{i}}$. These initial features represent the intrinsic attributes of each node prior to any encoding or learning process. In practical applications, such as in social networks, these features may include user-specific information like basic account details, gender, location, and follower count [2]. In contrast, for synthetic networks, initial features are often derived from structural metrics such as clustering coefficients, degree centrality, or other topological measures that capture the network’s connectivity and community structure. The quantum circuit for the mapping of node *v* is shown in Figure 4.
  In this circuit, the input is the quantum state |0〉. $R_{X}$ represents the rotation gate around the *X* axis in the quantum circuit. $x_{i_{k}}$ and $\theta_{k}$ denote the *k*-th component of the initial feature of node *i* and the *k*-th component of the initial rotation parameter, respectively. The output is the quantum state mapping $|\varphi_{i}\rangle$ of node *i*.
(2) Calculate the Euclidean distance correlation matrix *D* for the graph as follows: let $\vec{x_{i}}$ represent the initial feature of node *i*. The similarity between nodes *i* and *j* is computed as [56]
  (11) $$D_{ij}=\frac{\langle \vec{x_{i}},\vec{x_{j}}\rangle }{∥\vec{x_{i}}∥\;∥\vec{x_{j}}∥},\;\mathrm{with}\;i\neq j,$$
  where 〈·,·〉 denotes the inner product.
(3) Calculate the Hilbert space distance correlation matrix $D^{\prime }$ based on quantum state mappings. Let $|\varphi_{i}\rangle$ denote the quantum state mapping of node *i*. The similarity between nodes *i* and *j*, where i≠j is in the Hilbert space, is given by
  (12) $$D_{ij}^{\prime }=\langle \varphi_{i}|\varphi_{j}\rangle ,\mathrm{with}\;i\neq j.$$
(4) Compute the loss to adjust the initial rotation parameter $\vec{\theta }$. Define the loss function as
  (13) $$L=\sum_{ij}|D_{ij}-D_{ij}^{\prime }|.$$
  Use the interpolation-based derivative-free optimization method UOBYQA [57] to determine the optimal rotation parameter vector $\vec{\theta }$ that minimizes the loss function.

### 3.2. Decoder

The decoder constructed a multi-layer parameterized quantum circuit to approximate the Q-function, mapping the processed quantum state representation to a node importance ranking vector. In reinforcement learning, the process consists of the environment state *S*, the actions *A* taken in response to the environment, and the rewards *R* obtained after taking the actions. In the node ranking problem, the quantum state representation of the residual network after each round of node removal was treated as the state *S*, the quantum state representations of the nodes to be removed were treated as the action *A*, and the reduction in the accumulated network connectivity (ANC) [58] after node removal was used as the reward *R*. The formula for calculating ANC is as follows:

(14) $$ANC\left(v_{1},v_{2},\cdots ,v_{N}\right)=\frac{1}{N}\sum_{k=1}^{N}\frac{\sigma \left(G\setminus \left\{v_{1},v_{2},\ldots ,v_{k}\right\}\right)}{\sigma (G)},$$

where *N* represents the number of nodes, $v_{i}$ denotes the *i*-th removed node, and σ is the connectivity function. In this paper, the primary function of σ is to measure the size of each connected component in the network, thereby providing a reliable quantitative basis for evaluating overall connectivity. Specifically, we define σ(G) as $\sigma \left(G\right)=\sum_{C_{i}\in G}\frac{\delta_{i}\left(\delta_{i}-1\right)}{2}$, where $C_{i}$ denotes the i−-th connected component of the graph G, and $\delta_{i}$ represents the number of nodes within $C_{i}$. Accordingly, the reward $R_{t}$ at time *t* can be derived as $R_{t}=ANC\left(v_{1},v_{2},\cdots ,v_{t-1}\right)-ANC\left(v_{1},v_{2},\cdots ,v_{t}\right)$.

**Figure 5:**
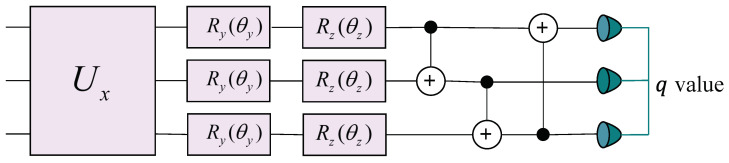
The quantum circuit diagram for a single layer of the decoder part. $U_{x}$ represents the data re-uploading, while the rotation angles of $R_{Y}$ and $R_{Z}$ are trainable parameters of the decoder. Each layer of rotation gates is succeeded by a layer of CNOT gates, facilitating entanglement within the system. The complete decoder is constructed by stacking multiple layers of this circuit.

To map the encoded quantum state representation |S〉 produced by the encoder into a Q-value for each node in the reinforcement learning framework, we constructed a multi-layer parameterized quantum circuit, as shown in Figure 5. Each layer consists of three primary operations:

1. Data re-uploading $U_{x}$, which re-uploads the state features onto the circuit [46,59].
2. Parameterized rotations $R_{y}\left(\theta_{y_{i}}\right)$ and $R_{z}\left(\theta_{z_{i}}\right)$, where $\left\{\theta_{y_{i}},\theta_{z_{i}}\right\}$ are trainable parameters corresponding to each qubit *i*. For the sake of clarity and conciseness, we denote the trainable parameters for the $R_{y}$ gates as $U_{y}$ and those for the $R_{z}$ gates as $U_{z}$. Collectively, $U_{y}$ and $U_{z}$ comprise the trainable parameter set of the decoder.
3. Entangling gates (CNOT), which entangle different qubits to capture correlations across the system.

Mathematically, we can represent one layer of the decoder circuit as follows:

(15) $$|y^{\left(l+1\right)}\rangle \;=\;\left(\prod_{j=1}^{J}{\mathrm{CNOT}}_{j}\right)\left(\overset{n}{\underset{i=1}{⨂}}U_{z}^{\left(l\right)}\;U_{y}^{\left(l\right)}\right)U_{x}\;|y^{\left(l\right)}\rangle ,$$

where $|y^{\left(l\right)}\rangle \in H^{⊗n}$ denotes the quantum state of the *n*-qubit system at the *l*-th decoder layer. The unitary operator $U_{x}$ performs data re-uploading, and the tensor product ${⨂}_{i=1}^{n}U_{z}^{\left(l\right)}U_{y}^{\left(l\right)}$ applies parameterized rotations about the *Y* and *Z* axes on each qubit. The subsequent ordered application of CNOT gates induces entanglement among the qubits.

The entanglement network implemented by the product of CNOT gates follows a ring topology, where each qubit acts as a control for the subsequent qubit, and the last qubit controls the first one. Specifically, for an *n*-qubit system, we implemented the sequence ${\mathrm{CNOT}}_{1\to 2}$, ${\mathrm{CNOT}}_{2\to 3}$, *…*, ${\mathrm{CNOT}}_{n-1\to n}$, ${\mathrm{CNOT}}_{n\to 1}$, where ${\mathrm{CNOT}}_{i\to j}$ indicates a CNOT gate with qubit *i* as the control and qubit *j* as the target. This circular arrangement ensures that information can propagate through the entire qubit register, enabling the creation of complex entangled states necessary for representing the Q-function.

Stacking *L* such layers yields the final state $|y^{\left(L\right)}\rangle$, and the trainable parameters $\left\{U_{y}^{\left(l\right)},U_{z}^{\left(l\right)}\right\}$ are optimized via repeated measurements to approximate the desired Q-function.

### 3.3. Computing Q-Values

Following the construction of the multi-layer decoder, projective measurements were performed on the final quantum state to extract Q-values. Let $|y^{\left(L\right)}\rangle$ denote the output state of the final decoder layer; the Q-value for an action $a_{t}$ in state $s_{t}$ can be expressed as the expectation value of a measurement operator ${\hat{M}}_{a_{t}}$:

(16) $$Q\left(s_{t},a_{t}\right)\;=\;\langle y^{\left(L\right)}|{\hat{M}}_{a_{t}}|y^{\left(L\right)}\rangle ,$$

where ${\hat{M}}_{a_{t}}$ is a Hermitian measurement operator chosen as the Pauli *Z* observable that corresponds to the Q-value of action $a_{t}$ [39]. Multiple measurement shots are employed to obtain a statistically robust estimate of the expectation value, which is used as the Q-value in the reinforcement learning procedure. Evaluating these Q-values for all feasible actions yields a ranking vector that reflects the relative importance of each node.

### 3.4. Loss Function Design

The trainable parameters consisted of two components: encoder parameters $\Theta_{E}=\left\{U_{1},U_{2}\right\}$ and decoder parameters $\Theta_{D}=\left\{U_{y},U_{z}\right\}$. The encoder error was measured by the quantum state representations of nodes after encoding, where connected nodes were expected to have similar quantum state features. The decoding error arose from the delayed update mechanism of deep Q-networks, where a target Q-network with an identical structure to Figure 5 but different parameters was constructed. The target Q-network’s initial parameters matched those of the Q-network updated at each step, with periodic updates from the Q-network parameters.

The Q-values generated after measuring the Q-network were denoted as $Q(s_{t},a_{t})$, where $s_{t}$ represents the environmental state at time *t*. The target Q-values produced by the target Q-network were expressed as $r_{t}+\gamma \max_{a}\;Q\left(s_{t+1},a\right)$, where $r_{t}$ represents the reward obtained at time *t*, and γ∈[0,1] is the discount factor weighing the importance of future rewards. Thus, the overall error for one training iteration was formulated as

(17) $$\begin{array}{cc} \mathrm{Loss}\left(\Theta_{E},\Theta_{D}\right) & =\alpha \sum_{i,j=1}^{N}s_{i,j}{∥|\varphi_{i}\rangle -|\varphi_{j}\rangle ;\Theta_{E\;}∥}_{2}^{2}\\ \; & +\;E_{\left(s_{t},a_{t},r_{t,t+n},s_{t+n}\right)∼U\left(B\right)}\left[{\left(r_{t,t+n}+\gamma \max_{a^{\prime }}\hat{Q}\left(s_{t+n},a^{\prime };{\hat{\Theta }}_{D}\right)-Q\left(s_{t},a_{t};\Theta_{D}\right)\right)}^{2}\right], \end{array}$$

where $E_{\left(s_{t},a_{t},r_{t,t+n},s_{t+n}\right)∼U\left(B\right)}$ represents the expectation value over samples randomly drawn from the replay memory to reduce sample correlation. The term $r_{t,t+n}$ denotes the *n*-step return, which is the accumulated reward from time step *t* to t+n. α denotes the encoding error weight. $s_{i,j}$ indicates whether node *i* and node *j* are connected. If i∈N(j), then $s_{i,j}=1$; otherwise, $s_{i,j}=0$. $|\varphi_{i}\rangle$ represents the quantum state feature of node *i* obtained after node encoding. $|\varphi_{i}\rangle$ represents the quantum state feature of node *i* obtained after node encoding. The semicolon notation indicates parametric dependence. The $\hat{Q}$ denotes the target network, which uses fixed parameters ${\hat{\Theta }}_{D}$ during optimization steps.

## 4. Experiments and Results

This model supports training on small synthetic networks and subsequent application in real-world scenarios. Synthetic networks were generated using the Erdős–Rényi (ER) [60] and the Watts–Strogatz (WS) [61] networks, with 30 to 50 nodes. For each node, initial feature representation is constructed from several topological metrics, including degree centrality [62], eigenvector centrality [63], betweenness centrality [64], closeness centrality [62], and the clustering coefficient [61]. Experimental results demonstrate that the model performed well when the network size was several hundred nodes. Comparative benchmarking against canonical centrality measures, including degree, PageRank [65], eigenvector, coreness [66], and betweenness centrality, confirms the feasibility of our proposed quantum-inspired algorithm. The results validate that the approach effectively operates within the established theoretical framework. The number of model parameters was linearly related to the number of network layers, thereby reducing computational complexity relative to classical deep neural networks, which often exhibit much faster parameter growth. In this section, we briefly describe the three empirical networks used in the experiments. Subsequently, we analyze the ranking results of QDRL on toy networks. Then, we illustrate the relationship between the node ranking performance of QDRL and the *p*-values in ER and WS networks.

### 4.1. Data Description

To evaluate the performance of QDRL, we performed experiments on three real-world networks:

Football [67]: This network, consisting of 115 nodes and 613 edges, represents the schedule of Division I college football games during the 2000 season in the United States. Each node corresponds to a football team, while each undirected edge indicates that a game was played between the two connected teams.

USAir [68]: The USAir network, consisting of 332 nodes and 2126 edges, represents the U.S. air transportation system. Each node corresponds to an airport, while each undirected edge indicates the existence of a direct flight connection between two airports.

Karate Club [69]: The Karate Club network, consisting of 34 nodes and 78 edges, represents the social relationships between members of a university karate club, as observed by Wayne Zachary in 1977. Each node corresponds to a club member, and each undirected edge indicates a social interaction or tie between two members.

Because the number of qubits available in current quantum hardware is limited, we selected relatively small networks to ensure the feasibility of our quantum circuit implementations. Despite their modest scale, these datasets exhibit diverse topological characteristics, thereby allowing us to assess the adaptability and robustness of our proposed method across different network structures.

### 4.2. QDRL Node Ranking on Real-World Datasets

**Figure 6:**
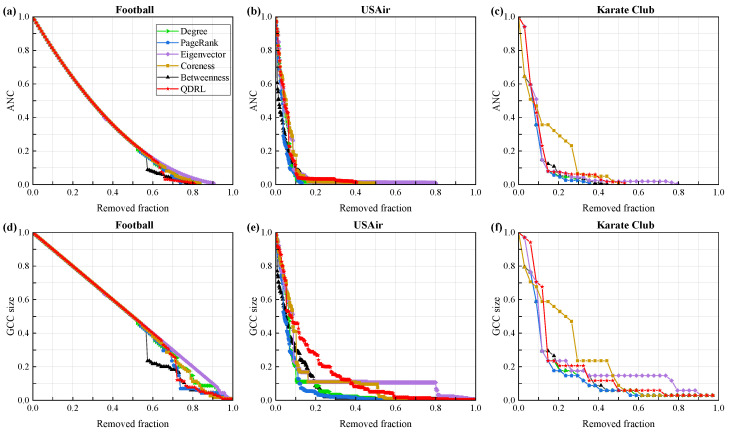
The dismantling performance of different methods on real-world networks. The x axis represents the proportion of nodes removed. In (**a**–**c**), the y axis denotes the ANC values after node removal, while in (**d**–**f**), the y axis represents the size of the GCC (giant connected component).

We compared our approach with node centrality metrics such as degree and betweenness, as well as the PageRank method, with the experimental results shown in Figure 6. Figure 6a–c evaluate the performance of the model from the perspective of the ANC curve. The results demonstrate that QDRL performed comparably to the baselines, particularly on smaller networks such as Football and Karate Club. Considering the limited number of qubits used during training, QDRL’s ability to aggregate information achieved an optimal state when applied to networks of smaller scale.

**Figure 7:**
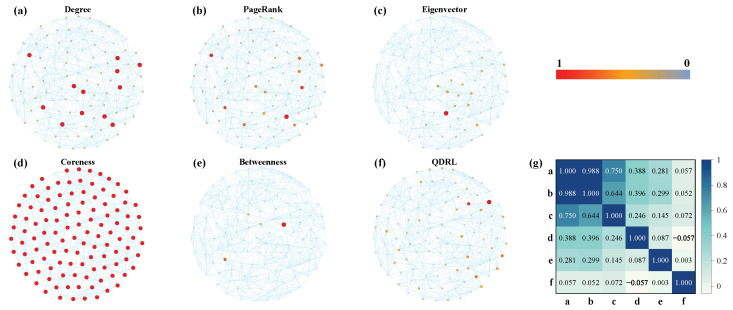
Visualization and correlation analysis of node rankings for six methods. Using the Football network as an example, the vertex colors in (**a**–**f**) are proportional to the normalized values of each method, applying min-max normalization. (**g**) visualizes the pairwise Pearson correlation, where the letters (**a**–**f**) on the x axis and y axis represent degree, PageRank, eigenvector, coreness, betweenness, and QDRL, respectively.

Figure 6a,d demonstrate that the trend lines of QDRL were similar to those of other methods. Therefore, we conducted a separate visualization and correlation analysis of node rankings for the Football dataset, as shown in Figure 7. Figure 7g presents the pairwise Pearson correlation coefficients computed over the rankings of all nodes. The Pearson correlation coefficient is defined as

(18) $$r_{xy}=\frac{\sum_{i=1}^{N}\left(x_{i}-\bar{x}\right)\left(y_{i}-\bar{y}\right)}{\sqrt{\sum_{i=1}^{N}{\left(x_{i}-\bar{x}\right)}^{2}}\sqrt{\sum_{i=1}^{N}{\left(y_{i}-\bar{y}\right)}^{2}}},$$

where $x_{i}$ and $y_{i}$ denote the ranking scores for node *i* from two different methods, and $\bar{x}$ and $\bar{y}$ are the corresponding mean values. A coefficient close to +1 indicates that the two methods yield similar ranking orders, whereas a coefficient close to −1 suggests that the rankings are inversely related. Values near zero indicate little or no linear correlation between the methods. This measure thus provides a quantitative assessment of the consistency between different node ranking approaches. Combined with the results from Figure 6 and Figure 7, it was evident that QDRL not only maintained comparable performance but also provided unique insights. Additionally, the QDRL approach identified influential nodes that were more uniformly distributed across the network topology, in contrast to traditional methods which tend to concentrate on densely connected regions. This spatial distribution of key nodes helped overcome the “rich-club” phenomenon, where importance is disproportionately assigned to highly interconnected nodes. The more balanced identification of influential nodes is particularly valuable for applications requiring diverse network coverage, such as information dissemination or network monitoring, as it prevents the overemphasis on already well-connected regions while recognizing important nodes in peripheral areas that might otherwise be overlooked.

**Figure 8:**
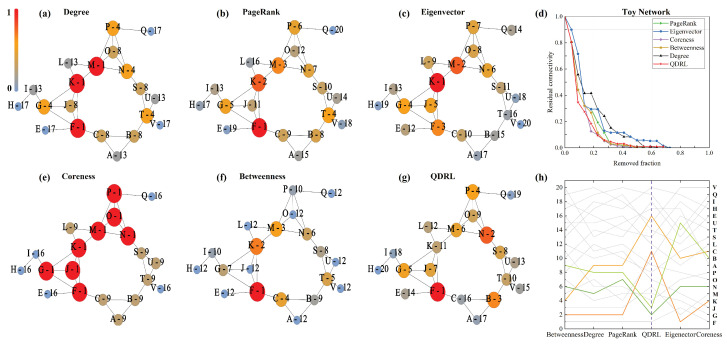
Node rankings in the toy network under six different methods. The specific rankings of each node are depicted in panels (**a**–**c**,**e**–**g**). The size and color of nodes are proportional to their perceived importance, with rank 1 representing the highest importance and rank 20 the lowest. Panel (**d**) shows the ANC curves of the six methods, with the x axis representing the proportion of nodes removed. Panel (**h**) shows the trend of ranking changes for each node across the different methods.

To further illustrate QDRL’s capability to mitigate the effects of localization, we constructed a toy network to demonstrate QDRL’s ability to capture global information, as shown in Figure 8. We selected two groups of node sets for analysis, each containing two nodes: {C,K} and {B,N}. Figure 8 reveals that nodes *C* and *K* are direct neighbors of node *F*, which is highly influential. While proximity to an influential node often increases a node’s ranking in classical centrality metrics, QDRL incorporates additional structural information that prevents overemphasis on immediate adjacency to a single dominant node. Specifically, QDRL learns from the global dismantling effect observed during training, thereby assigning lower ranks to *C* and *K* despite their closeness to *F*. This does not imply that *F* should have no influence at all but rather that QDRL balances local proximity with broader connectivity patterns. Consequently, *C* and *K* receive rankings that diverge from those assigned by other methods, underscoring QDRL’s capacity to capture network-wide context.

In contrast, nodes *B* and *N* receive higher rankings under QDRL. Although these nodes do not have particularly high degrees, Figure 8g shows that they act as structural “bridges”, connecting multiple substructures in the network. Removing *B* or *N* increases fragmentation more effectively than might be suggested by local metrics alone. It should be noted that other nodes such as *S* or *K* can also exhibit bridging properties in certain contexts. However, in this particular configuration, *B* and *N* play a more critical role in the global dismantling process, leading QDRL to rank them among the top three nodes alongside *F*. After removing *F*, *N*, and *B*, as identified by QDRL, the network splits into more evenly distributed subgraphs compared to other methods, indicating a reduction in the localization of message propagation.

### 4.3. QDRL Node Ranking on Synthetic Networks with Varying Edge Densities

**Figure 9:**
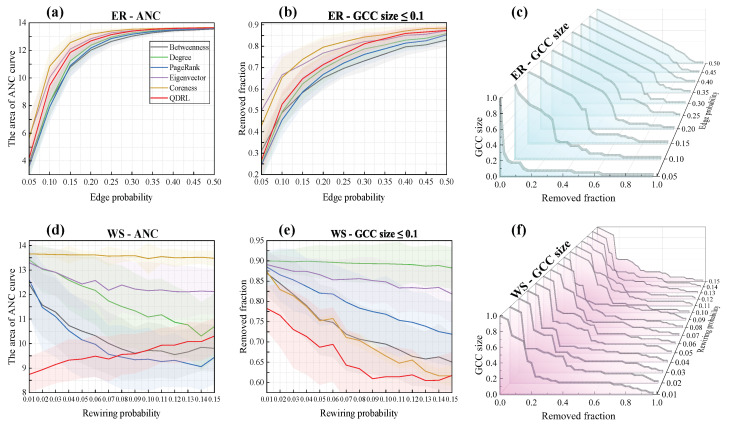
A performance comparison of six methods under varying edge densities on synthetic graphs generated using ER and WS networks. Panel (**a**,**d**) show the area under the ANC curves for each generated network. Panel (**b**,**e**) depict the proportion of nodes removed when the GCC size was 10% of the total network size. Panel (**c**,**f**) illustrate the curves of the optimal GCC size for each corresponding *p*-value.

To investigate the relationship between QDRL’s performance and network properties, we conducted analyses based on the ER [60] and WS [61] networks, with the generated networks set to a size of n=40. For th ER networks, the edge probability $p_{ER}$-value was in the range [0.05, 0.5] with an interval of 0.05, while for the WS networks, the initial node degree *k* was fixed at 4, and the rewiring probability $p_{WS}$-value was in the range [0.01, 0.15] with an interval of 0.01. For each $p_{ER}$-value and $p_{WS}$-value, 100 networks were generated. The results are shown in Figure 9.

From Figure 9, the area under the ANC curve reflects the speed of network disintegration and the degree of fragmentation. A smaller area indicates faster disintegration and more fragmented connected components, suggesting that node removal has a greater destructive impact on the network. In contrast, a larger area signifies slower disintegration and greater resistance to fragmentation attacks, implying that the identified nodes do not serve as critical bridging nodes in the network. In Figure 9a,d, it can be observed that QDRL performed comparably to other methods in the ER networks. As shown in Figure 9c, when $p_{ER}$ was small, network disintegration occurred more rapidly, whereas for larger $p_{ER}$-values, the network dismantling rate initially decreased gradually and then accelerated. This is attributed to the increased homogeneity of information in strongly connected networks. In the WS networks, as illustrated in Figure 9d,e, QDRL significantly outperformed other methods when $p_{WS}$ was small, indicating QDRL’s ability to identify more influential nodes in highly clustered topologies. Although the clustering coefficient remained relatively high and did not vary significantly within the range $0.01<p_{WS}<0.15$, the average path length decreased noticeably as $p_{WS}$ increased. This reduction in path length implies that nodes become more globally interconnected, thereby influencing how QDRL ranks nodes in terms of their overall impact on network disintegration. As shown in Figure 9e, even as $p_{WS}$ grew, the GCC size under QDRL remained markedly lower than for other methods, suggesting that QDRL captures these long-range dependencies effectively and disperses network fragments more evenly. Moreover, Figure 9f shows that QDRL’s disintegration trend line exhibited reduced fluctuation compared to Figure 9c, reflecting that QDRL strikes a better balance between regularity and randomness when determining node influence rankings in WS networks.

## 5. Conclusions

In this study, we proposed a quantum deep reinforcement learning algorithm for identifying critical nodes in networks, thereby demonstrating that network analysis tasks can be effectively addressed with quantum algorithms. The model was designed within a reinforcement learning framework, comprising an encoder and a decoder. The encoder utilized Quantum GraphSage to aggregate multi-hop neighbor information, capture long-range correlations between nodes, and preserve the original graph structure. The decoder employed a quantum DDQN to ensure stability during model training. This model was trained on small synthetic networks and subsequently applied to real-world networks, demonstrating its superior generalization performance. Benefiting from quantum computing, the parameter count of the model scaled linearly with the number of network layers, significantly improving training efficiency.

The experimental results indicate that QDRL achieves performance comparable to classical methods on small-scale networks, serving as a proof-of-concept for the applicability of quantum approaches to network analysis. Given the current limitations in available qubits, our experiments were conducted on relatively small networks; however, these findings reveal that quantum algorithms can offer unique insights into node importance rankings and network dynamics.

Looking ahead, as quantum computing technology advances and larger quantum systems become accessible, the scalability and computational advantages of our approach are expected to increase. Future quantum processors, with their inherent parallelism and exponential state space, could enable our model to be applied to much larger and more complex networks, ultimately leading to superior performance compared to classical techniques.

Overall, our work establishes a foundation for leveraging quantum deep reinforcement learning in complex network analysis and paves the way for further exploration as quantum hardware continues to evolve.

## Data Availability

The authors declare that the code and data supporting the findings of this study will be available after this paper is published at the following GitHub repository: https://github.com/Katherine-Gilber/QDRL.
